# Supplementary figures and images for: Identification of Candidate Ice Nucleation Activity (INA) Genes in Fusarium avenaceum by Combining Phenotypic Characterization with Comparative Genomics and Transcriptomics
Source: J Fungi (Basel). 2022 Sep 13;8(9):958. doi: 10.3390/jof8090958 (PMC9501429; doi:10.3390/jof8090958)

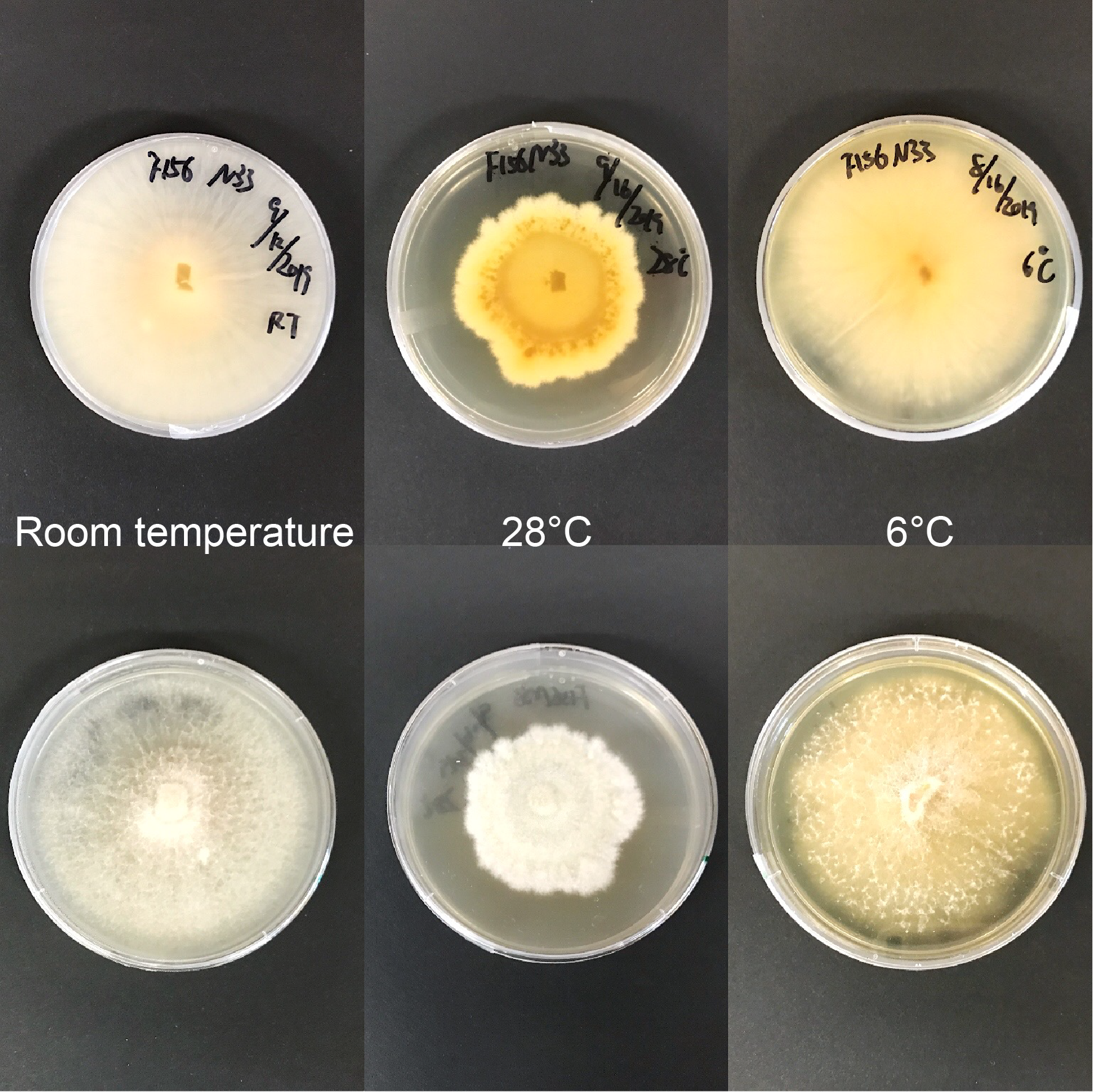

Supplement: Supplementary file 1 [file jof-08-00958-s001.zip › Supplementary_1_FigureS1.png]
